# Cyclo­pentyl­diphen­yl(4-thio­semi­carbazonopenta­noato-κ*O*)tin(IV)

**DOI:** 10.1107/S1600536808011112

**Published:** 2008-04-26

**Authors:** Kong Mun Lo, Seik Weng Ng

**Affiliations:** a Department of Chemistry, University of Malaya, 50603 Kuala Lumpur, Malaysia

## Abstract

The Sn atom in the title compound, [Sn(C_5_H_9_)(C_6_H_5_)_2_(C_6_H_10_N_3_O_2_S)], exists within a tetra­hedral geometry. The –NH_2_ group forms a weak hydrogen bond across a center of inversion to the S atom of an adjacent mol­ecule, as well as another weaker hydrogen (across another center of inversion) to the Sn-bound O atom of another mol­ecule. The hydrogen-bonded layer structure is consolidated by a strong hydrogen bond between the –NH– group and the uncoordinated O atom of a third mol­ecule.

## Related literature

For the antibacterial and antifungal applications of cyclo­pentyl­diphenyl­tin carboxyl­ates, see: Koshy *et al.* (2001 ▶). For the crystal structures of cyclo­pentyl­diphenyl­tin derivatives, see: Lo & Ng (2004 ▶); Lo *et al.* (1999 ▶); Teo *et al.* (2004 ▶). For the synthesis of levulinic acid thio­semicarbazone, see: Ng (1992 ▶). For a review of the structural chemistry of organotin carboxyl­ates, see: Tiekink (1991 ▶, 1994 ▶).
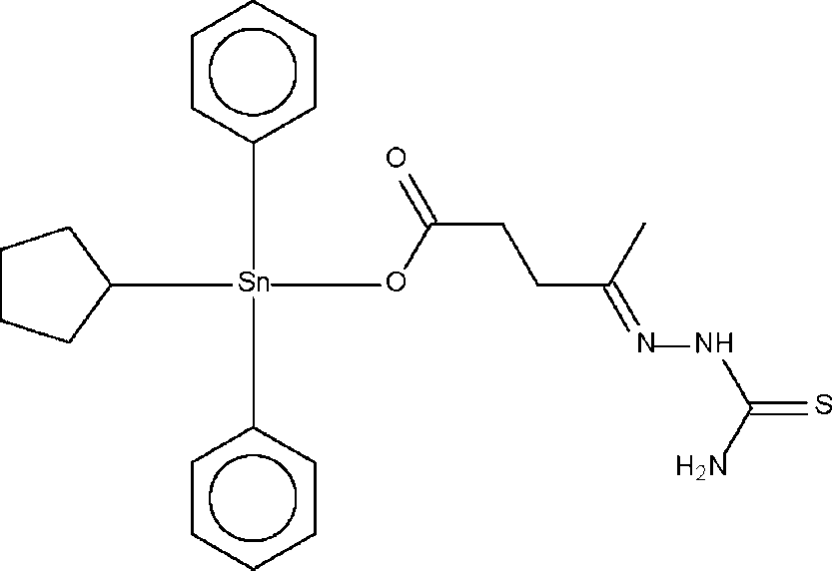


## Experimental

### 

#### Crystal data

[Sn(C_5_H_9_)(C_6_H_5_)_2_(C_6_H_10_N_3_O_2_S)]
*M* *_r_* = 530.24
Triclinic, 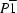

*a* = 9.5780 (1) Å
*b* = 10.2375 (1) Å
*c* = 13.4205 (1) Å
α = 86.901 (1)°
β = 83.370 (1)°
γ = 63.667 (1)°
*V* = 1171.50 (2) Å^3^
*Z* = 2
Mo *K*α radiation
μ = 1.20 mm^−1^
*T* = 100 (2) K
0.30 × 0.15 × 0.10 mm

#### Data collection

Bruker SMART APEX diffractometer
Absorption correction: multi-scan (*SADABS*; Sheldrick, 1996 ▶) *T*_min_ = 0.779, *T*_max_ = 0.889
15020 measured reflections
5350 independent reflections
5186 reflections with *I* > 2σ(*I*)
*R*_int_ = 0.014

#### Refinement

*R*[*F* ^2^ > 2σ(*F* ^2^)] = 0.028
*wR*(*F* ^2^) = 0.078
*S* = 1.03
5350 reflections
272 parameters
H-atom parameters constrained
Δρ_max_ = 1.99 e Å^−3^
Δρ_min_ = −0.91 e Å^−3^

### 

Data collection: *APEX2* (Bruker, 2007 ▶); cell refinement: *SAINT* (Bruker, 2007 ▶); data reduction: *SAINT*; program(s) used to solve structure: *SHELXS97* (Sheldrick, 2008 ▶); program(s) used to refine structure: *SHELXL97* (Sheldrick, 2008 ▶); molecular graphics: *X-SEED* (Barbour, 2001 ▶); software used to prepare material for publication: *publCIF* (Westrip, 2008 ▶).

## Supplementary Material

Crystal structure: contains datablocks global, I. DOI: 10.1107/S1600536808011112/tk2255sup1.cif

Structure factors: contains datablocks I. DOI: 10.1107/S1600536808011112/tk2255Isup2.hkl

Additional supplementary materials:  crystallographic information; 3D view; checkCIF report

## Figures and Tables

**Table:** 

Sn1—O1	2.063 (2)
Sn1—C1	2.131 (3)
Sn1—C6	2.125 (2)
Sn1—C12	2.134 (2)

**Table:** 

O1—Sn1—C1	112.7 (1)
O1—Sn1—C6	108.6 (1)
O1—Sn1—C12	95.9 (1)
C1—Sn1—C6	116.5 (1)
C1—Sn1—C12	112.1 (1)
C6—Sn1—C12	109.2 (1)
Sn1—O1—C18	109.3 (1)

**Table 2:** Hydrogen-bond geometry (Å, °)

*D*—H⋯*A*	*D*—H	H⋯*A*	*D*⋯*A*	*D*—H⋯*A*
N2—H2*n*⋯O2^i^	0.88	2.12	2.975 (3)	163
N3—H3*n*1⋯O1^ii^	0.88	2.43	3.121 (3)	136
N3—H3*n*2⋯S1^iii^	0.88	2.54	3.389 (2)	161

Symmetry codes: (i) 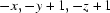; (ii) 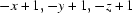; (iii) 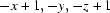.

**Fig. 1.**
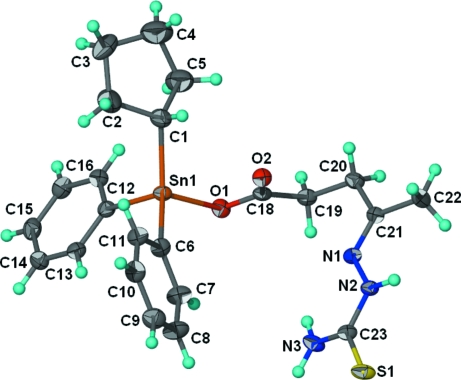
70% Probability thermal ellipsoid plot of Sn(C5H9)(C6H5)2(C12H15N3O2S), (I), show atom-numbering scheme. Hydrogen atoms are drawn as spheres of arbitrary radius.

## supplementary crystallographic information

### Comment

Triorganotin carboxylates having two different organyl substituents possess ehanced anti-bacterial and anti-fungal properties compared with the symmetrical compounds (Koshy *et al.*, 2001). The synthesis of cyclopentyldiphenyltin hydroxide, which is the principal reagent that condenses readily with carboxylic acids, is a multi-step synthesis. Previous studies have characterized a few cyclopentyldiphenyltin derivatives (Lo & Ng, 2004; Lo *et al.*, 1999; Teo *et al.*, 2004). In the reaction with levulinic acid thiosemicarbazone (Ng, 1992), the organotin hydroxide yields a four-coordinate compound (I) (Fig. 1 & Table 1). The tin atom exists in a tetrahedral geometry; adjacent molecules are linked by hydrogen bonds into a layer structure, Table 2.

### Experimental

Levulinic acid thiosemicarbazone was synthesized from the reaction of levulinic acid and thiosemicarbazide (Ng, 1992). Cyclopentyldiphenyltin hydroxide was sythesized by using a multistep reaction, starting from the Grignard reaction of cyclopentylmagnesium bromide on triphenyltin chloride. One phenyl radical was then cleaved by iodine in DMF; the resulting iodide was then hydrolyzed with sodium hydroxide in acetone to give the mixed triorganotin hydroxide (Lo *et al.*, 1999). The thiosemicarbazone (1.1 g, 5 mmol) and triorganotin hydroxide (2 g, 5 mmol) were dissolved in hot ethanol (50 ml). The clear solution was filtered and colorless crystals separated from the cool solution after a day (yield: 75%).

### Refinement

Carbon-bound H-atoms were placed in calculated positions (C—H 0.95 to 0.99 Å) and were included in the refinement in the riding model approximation, with *U*(H) set to 1.2 to 1.5*U*(C). The nitrogen-bound H-atom were similarly generated (N–H 0.88±0.01 Å) and their temperature factors similarly tied.

The final difference Fourier map had a large peak at 1.4 Å from C1 but was otherwise diffuse.

### Crystal data

**Table:**

[Sn(C_5_H_9_)(C_6_H_5_)_2_(C_6_H_10_N_3_O_2_S)]	*Z* = 2
*M**_r_* = 530.24	*F*_000_ = 540
Triclinic, *P*1	*D*_x_ = 1.503 Mg m^−^^3^
Hall symbol: -P 1	Mo *K*α radiation λ = 0.71073 Å
*a* = 9.5780 (1) Å	Cell parameters from 9905 reflections
*b* = 10.2375 (1) Å	θ = 2.4–28.3º
*c* = 13.4205 (1) Å	µ = 1.20 mm^−^^1^
α = 86.901 (1)º	*T* = 100 (2) K
β = 83.370 (1)º	Irregular block, colorless
γ = 63.667 (1)º	0.30 × 0.15 × 0.10 mm
*V* = 1171.50 (2) Å^3^	

### Data collection

**Table:**

Bruker SMART APEXII diffractometer	5350 independent reflections
Radiation source: fine-focus sealed tube	5186 reflections with *I* > 2σ(*I*)
Monochromator: graphite	*R*_int_ = 0.014
*T* = 100(2) K	θ_max_ = 27.5º
ω scans	θ_min_ = 1.5º
Absorption correction: multi-scan(SADABS; Sheldrick, 1996)	*h* = −9→12
*T*_min_ = 0.779, *T*_max_ = 0.889	*k* = −13→13
15020 measured reflections	*l* = −17→17

### Refinement

**Table:**

Refinement on *F*^2^	Secondary atom site location: difference Fourier map
Least-squares matrix: full	Hydrogen site location: inferred from neighbouring sites
*R*[*F*^2^ > 2σ(*F*^2^)] = 0.028	H-atom parameters constrained
*wR*(*F*^2^) = 0.078	*w* = 1/[σ^2^(*F*_o_^2^) + (0.0431*P*)^2^ + 2.3757*P*] where *P* = (*F*_o_^2^ + 2*F*_c_^2^)/3
*S* = 1.03	(Δ/σ)_max_ < 0.001
5350 reflections	Δρ_max_ = 1.99 e Å^−^^3^
272 parameters	Δρ_min_ = −0.91 e Å^−^^3^
Primary atom site location: structure-invariant direct methods	Extinction correction: none

### Fractional atomic coordinates and isotropic or equivalent isotropic displacement parameters (Å^2^)

**Table:**

	*x*	*y*	*z*	*U*_iso_*/*U*_eq_	
Sn1	0.400900 (17)	0.628156 (16)	0.232609 (11)	0.01648 (6)	
S1	0.24404 (7)	0.05406 (6)	0.56139 (5)	0.02291 (13)	
O1	0.4075 (2)	0.63541 (19)	0.38521 (13)	0.0191 (3)	
O2	0.1648 (2)	0.66366 (19)	0.38475 (13)	0.0188 (3)	
N1	0.1747 (2)	0.4584 (2)	0.57946 (15)	0.0161 (4)	
N2	0.1483 (2)	0.3354 (2)	0.58783 (15)	0.0160 (4)	
H2N	0.0552	0.3410	0.6087	0.019*	
N3	0.4100 (2)	0.2066 (2)	0.54086 (17)	0.0215 (4)	
H3N1	0.4187	0.2883	0.5433	0.026*	
H3N2	0.4933	0.1248	0.5240	0.026*	
C1	0.2123 (3)	0.8151 (3)	0.1783 (2)	0.0269 (5)	
H1	0.1189	0.7944	0.1863	0.032*	
C2	0.2415 (5)	0.8439 (5)	0.0673 (3)	0.0712 (16)	
H2A	0.1932	0.8001	0.0267	0.085*	
H2B	0.3553	0.8010	0.0460	0.085*	
C3	0.1675 (4)	1.0097 (4)	0.0531 (2)	0.0359 (7)	
H3A	0.2491	1.0442	0.0376	0.043*	
H3B	0.0987	1.0397	−0.0019	0.043*	
C4	0.0732 (5)	1.0696 (4)	0.1530 (3)	0.0495 (9)	
H4A	−0.0343	1.0788	0.1542	0.059*	
H4B	0.0672	1.1663	0.1663	0.059*	
C5	0.1651 (4)	0.9556 (3)	0.2307 (3)	0.0398 (7)	
H5A	0.2577	0.9674	0.2456	0.048*	
H5B	0.0978	0.9625	0.2940	0.048*	
C6	0.4168 (3)	0.4221 (3)	0.19650 (18)	0.0199 (5)	
C7	0.4926 (3)	0.2975 (3)	0.2541 (2)	0.0245 (5)	
H7	0.5361	0.3044	0.3126	0.029*	
C8	0.5049 (3)	0.1635 (3)	0.2263 (2)	0.0285 (6)	
H8	0.5560	0.0794	0.2661	0.034*	
C9	0.4429 (3)	0.1523 (3)	0.1411 (2)	0.0271 (5)	
H9	0.4516	0.0607	0.1223	0.032*	
C10	0.3684 (3)	0.2743 (3)	0.08321 (19)	0.0238 (5)	
H10	0.3244	0.2668	0.0251	0.029*	
C11	0.3575 (3)	0.4079 (3)	0.10956 (19)	0.0225 (5)	
H11	0.3093	0.4906	0.0680	0.027*	
C12	0.6217 (3)	0.6303 (3)	0.19063 (17)	0.0176 (4)	
C13	0.7494 (3)	0.5069 (3)	0.14887 (18)	0.0201 (5)	
H13	0.7382	0.4206	0.1403	0.024*	
C14	0.8925 (3)	0.5089 (3)	0.11974 (19)	0.0236 (5)	
H14	0.9784	0.4245	0.0913	0.028*	
C15	0.9095 (3)	0.6343 (3)	0.13229 (19)	0.0243 (5)	
H15	1.0070	0.6359	0.1118	0.029*	
C16	0.7847 (3)	0.7577 (3)	0.17470 (19)	0.0231 (5)	
H16	0.7970	0.8432	0.1841	0.028*	
C17	0.6419 (3)	0.7553 (3)	0.20323 (18)	0.0198 (5)	
H17	0.5565	0.8400	0.2318	0.024*	
C18	0.2692 (3)	0.6595 (2)	0.43182 (18)	0.0163 (4)	
C19	0.2537 (3)	0.6844 (3)	0.54288 (18)	0.0184 (4)	
H19A	0.2739	0.7692	0.5536	0.022*	
H19B	0.3354	0.5982	0.5733	0.022*	
C20	0.0950 (3)	0.7117 (3)	0.59732 (18)	0.0194 (5)	
H20A	0.0866	0.7515	0.6645	0.023*	
H20B	0.0126	0.7872	0.5600	0.023*	
C21	0.0628 (3)	0.5805 (2)	0.61014 (17)	0.0162 (4)	
C22	−0.0961 (3)	0.6051 (3)	0.65817 (19)	0.0211 (5)	
H22A	−0.1493	0.7023	0.6884	0.032*	
H22B	−0.0851	0.5313	0.7103	0.032*	
H22C	−0.1579	0.5979	0.6073	0.032*	
C23	0.2715 (3)	0.2065 (2)	0.56254 (17)	0.0170 (4)	

### Atomic displacement parameters (Å^2^)

**Table:**

	*U*^11^	*U*^22^	*U*^33^	*U*^12^	*U*^13^	*U*^23^
Sn1	0.01453 (9)	0.01349 (9)	0.02213 (10)	−0.00650 (6)	−0.00357 (6)	0.00085 (6)
S1	0.0168 (3)	0.0119 (3)	0.0404 (4)	−0.0064 (2)	−0.0043 (2)	0.0007 (2)
O1	0.0150 (8)	0.0209 (8)	0.0235 (8)	−0.0098 (7)	−0.0020 (6)	−0.0008 (6)
O2	0.0167 (8)	0.0197 (8)	0.0223 (8)	−0.0099 (7)	−0.0036 (6)	0.0004 (6)
N1	0.0180 (9)	0.0135 (9)	0.0185 (9)	−0.0081 (8)	−0.0033 (7)	−0.0001 (7)
N2	0.0139 (9)	0.0128 (9)	0.0219 (9)	−0.0065 (7)	−0.0021 (7)	0.0007 (7)
N3	0.0148 (9)	0.0140 (9)	0.0349 (11)	−0.0058 (8)	0.0000 (8)	−0.0047 (8)
C1	0.0213 (12)	0.0260 (13)	0.0251 (12)	−0.0028 (10)	−0.0042 (10)	0.0019 (10)
C2	0.046 (2)	0.068 (3)	0.040 (2)	0.023 (2)	0.0084 (16)	0.0231 (19)
C3	0.0378 (16)	0.0442 (18)	0.0334 (15)	−0.0241 (14)	−0.0144 (12)	0.0166 (13)
C4	0.049 (2)	0.0292 (16)	0.064 (2)	−0.0123 (15)	−0.0080 (18)	0.0046 (15)
C5	0.0435 (18)	0.0289 (15)	0.0423 (17)	−0.0111 (14)	−0.0067 (14)	0.0004 (13)
C6	0.0214 (11)	0.0190 (11)	0.0228 (11)	−0.0120 (10)	−0.0032 (9)	0.0000 (9)
C7	0.0274 (13)	0.0209 (12)	0.0287 (13)	−0.0120 (10)	−0.0120 (10)	0.0026 (10)
C8	0.0327 (14)	0.0183 (12)	0.0366 (14)	−0.0120 (11)	−0.0105 (11)	0.0048 (10)
C9	0.0285 (14)	0.0221 (12)	0.0323 (14)	−0.0124 (11)	−0.0035 (11)	−0.0021 (10)
C10	0.0257 (13)	0.0257 (13)	0.0224 (12)	−0.0133 (11)	−0.0020 (10)	−0.0032 (9)
C11	0.0236 (12)	0.0205 (12)	0.0230 (12)	−0.0091 (10)	−0.0052 (9)	0.0021 (9)
C12	0.0163 (11)	0.0177 (11)	0.0186 (10)	−0.0072 (9)	−0.0035 (8)	0.0027 (8)
C13	0.0215 (12)	0.0169 (11)	0.0209 (11)	−0.0075 (9)	−0.0030 (9)	0.0021 (9)
C14	0.0188 (12)	0.0238 (12)	0.0227 (12)	−0.0050 (10)	−0.0008 (9)	0.0009 (9)
C15	0.0200 (12)	0.0322 (14)	0.0232 (12)	−0.0144 (11)	−0.0020 (9)	0.0040 (10)
C16	0.0265 (13)	0.0260 (12)	0.0233 (12)	−0.0171 (11)	−0.0041 (10)	0.0015 (9)
C17	0.0194 (11)	0.0185 (11)	0.0211 (11)	−0.0079 (9)	−0.0026 (9)	−0.0003 (9)
C18	0.0165 (11)	0.0098 (9)	0.0234 (11)	−0.0063 (8)	−0.0023 (8)	−0.0002 (8)
C19	0.0206 (11)	0.0142 (10)	0.0228 (11)	−0.0095 (9)	−0.0038 (9)	−0.0014 (8)
C20	0.0202 (11)	0.0143 (10)	0.0226 (11)	−0.0064 (9)	−0.0013 (9)	−0.0029 (8)
C21	0.0163 (11)	0.0157 (10)	0.0162 (10)	−0.0063 (9)	−0.0033 (8)	−0.0009 (8)
C22	0.0172 (11)	0.0209 (11)	0.0234 (11)	−0.0070 (9)	−0.0006 (9)	−0.0032 (9)
C23	0.0173 (11)	0.0139 (10)	0.0203 (11)	−0.0068 (9)	−0.0050 (8)	0.0006 (8)

### Geometric parameters (Å, °)

**Table:**

Sn1—O1	2.063 (2)	C7—H7	0.9500
Sn1—C1	2.131 (3)	C8—C9	1.379 (4)
Sn1—C6	2.125 (2)	C8—H8	0.9500
Sn1—C12	2.134 (2)	C9—C10	1.380 (4)
S1—C23	1.695 (2)	C9—H9	0.9500
O1—C18	1.320 (3)	C10—C11	1.387 (4)
O2—C18	1.227 (3)	C10—H10	0.9500
N1—C21	1.283 (3)	C11—H11	0.9500
N1—N2	1.387 (3)	C12—C13	1.398 (3)
N2—C23	1.350 (3)	C12—C17	1.399 (3)
N2—H2N	0.8800	C13—C14	1.391 (4)
N3—C23	1.325 (3)	C13—H13	0.9500
N3—H3N1	0.8800	C14—C15	1.384 (4)
N3—H3N2	0.8800	C14—H14	0.9500
C1—C5	1.492 (4)	C15—C16	1.389 (4)
C1—C2	1.522 (4)	C15—H15	0.9500
C1—H1	1.0000	C16—C17	1.388 (4)
C2—C3	1.532 (5)	C16—H16	0.9500
C2—H2A	0.9900	C17—H17	0.9500
C2—H2B	0.9900	C18—C19	1.505 (3)
C3—C4	1.519 (5)	C19—C20	1.520 (3)
C3—H3A	0.9900	C19—H19A	0.9900
C3—H3B	0.9900	C19—H19B	0.9900
C4—C5	1.547 (5)	C20—C21	1.503 (3)
C4—H4A	0.9900	C20—H20A	0.9900
C4—H4B	0.9900	C20—H20B	0.9900
C5—H5A	0.9900	C21—C22	1.499 (3)
C5—H5B	0.9900	C22—H22A	0.9800
C6—C11	1.396 (3)	C22—H22B	0.9800
C6—C7	1.398 (3)	C22—H22C	0.9800
C7—C8	1.393 (4)		
			
O1—Sn1—C1	112.7 (1)	C8—C9—H9	120.0
O1—Sn1—C6	108.6 (1)	C10—C9—H9	120.0
O1—Sn1—C12	95.9 (1)	C9—C10—C11	120.2 (2)
C1—Sn1—C6	116.5 (1)	C9—C10—H10	119.9
C1—Sn1—C12	112.1 (1)	C11—C10—H10	119.9
C6—Sn1—C12	109.2 (1)	C10—C11—C6	120.7 (2)
Sn1—O1—C18	109.3 (1)	C10—C11—H11	119.7
C21—N1—N2	118.3 (2)	C6—C11—H11	119.7
C23—N2—N1	117.11 (19)	C13—C12—C17	118.4 (2)
C23—N2—H2N	121.4	C13—C12—Sn1	120.71 (18)
N1—N2—H2N	121.4	C17—C12—Sn1	120.94 (18)
C23—N3—H3N1	120.0	C14—C13—C12	120.8 (2)
C23—N3—H3N2	120.0	C14—C13—H13	119.6
H3N1—N3—H3N2	120.0	C12—C13—H13	119.6
C5—C1—C2	106.2 (3)	C15—C14—C13	119.9 (2)
C5—C1—Sn1	116.7 (2)	C15—C14—H14	120.1
C2—C1—Sn1	113.0 (2)	C13—C14—H14	120.1
C5—C1—H1	106.8	C14—C15—C16	120.4 (2)
C2—C1—H1	106.8	C14—C15—H15	119.8
Sn1—C1—H1	106.8	C16—C15—H15	119.8
C1—C2—C3	106.9 (3)	C17—C16—C15	119.5 (2)
C1—C2—H2A	110.3	C17—C16—H16	120.2
C3—C2—H2A	110.3	C15—C16—H16	120.2
C1—C2—H2B	110.3	C16—C17—C12	121.1 (2)
C3—C2—H2B	110.3	C16—C17—H17	119.5
H2A—C2—H2B	108.6	C12—C17—H17	119.5
C4—C3—C2	104.5 (3)	O2—C18—O1	120.5 (2)
C4—C3—H3A	110.9	O2—C18—C19	125.2 (2)
C2—C3—H3A	110.9	O1—C18—C19	114.2 (2)
C4—C3—H3B	110.9	C18—C19—C20	114.6 (2)
C2—C3—H3B	110.9	C18—C19—H19A	108.6
H3A—C3—H3B	108.9	C20—C19—H19A	108.6
C3—C4—C5	104.1 (3)	C18—C19—H19B	108.6
C3—C4—H4A	110.9	C20—C19—H19B	108.6
C5—C4—H4A	110.9	H19A—C19—H19B	107.6
C3—C4—H4B	110.9	C21—C20—C19	115.40 (19)
C5—C4—H4B	110.9	C21—C20—H20A	108.4
H4A—C4—H4B	109.0	C19—C20—H20A	108.4
C1—C5—C4	102.4 (3)	C21—C20—H20B	108.4
C1—C5—H5A	111.3	C19—C20—H20B	108.4
C4—C5—H5A	111.3	H20A—C20—H20B	107.5
C1—C5—H5B	111.3	N1—C21—C22	126.5 (2)
C4—C5—H5B	111.3	N1—C21—C20	116.6 (2)
H5A—C5—H5B	109.2	C22—C21—C20	116.8 (2)
C11—C6—C7	118.4 (2)	C21—C22—H22A	109.5
C11—C6—Sn1	119.38 (18)	C21—C22—H22B	109.5
C7—C6—Sn1	122.09 (18)	H22A—C22—H22B	109.5
C8—C7—C6	120.4 (2)	C21—C22—H22C	109.5
C8—C7—H7	119.8	H22A—C22—H22C	109.5
C6—C7—H7	119.8	H22B—C22—H22C	109.5
C9—C8—C7	120.2 (3)	N3—C23—N2	117.2 (2)
C9—C8—H8	119.9	N3—C23—S1	123.29 (18)
C7—C8—H8	119.9	N2—C23—S1	119.54 (18)
C8—C9—C10	120.0 (2)		
			
C6—Sn1—O1—C18	−77.70 (16)	C9—C10—C11—C6	−2.2 (4)
C1—Sn1—O1—C18	52.80 (17)	C7—C6—C11—C10	2.5 (4)
C12—Sn1—O1—C18	169.72 (15)	Sn1—C6—C11—C10	179.0 (2)
C21—N1—N2—C23	−174.8 (2)	O1—Sn1—C12—C13	110.54 (19)
O1—Sn1—C1—C5	32.7 (3)	C6—Sn1—C12—C13	−1.5 (2)
C6—Sn1—C1—C5	159.1 (2)	C1—Sn1—C12—C13	−132.09 (19)
C12—Sn1—C1—C5	−74.2 (2)	O1—Sn1—C12—C17	−70.09 (19)
O1—Sn1—C1—C2	156.3 (3)	C6—Sn1—C12—C17	177.87 (18)
C6—Sn1—C1—C2	−77.3 (3)	C1—Sn1—C12—C17	47.3 (2)
C12—Sn1—C1—C2	49.4 (3)	C17—C12—C13—C14	−0.6 (4)
C5—C1—C2—C3	−15.5 (4)	Sn1—C12—C13—C14	178.79 (18)
Sn1—C1—C2—C3	−144.7 (3)	C12—C13—C14—C15	0.2 (4)
C1—C2—C3—C4	−9.9 (5)	C13—C14—C15—C16	0.6 (4)
C2—C3—C4—C5	30.7 (4)	C14—C15—C16—C17	−0.9 (4)
C2—C1—C5—C4	34.0 (4)	C15—C16—C17—C12	0.4 (4)
Sn1—C1—C5—C4	161.0 (2)	C13—C12—C17—C16	0.3 (4)
C3—C4—C5—C1	−40.3 (4)	Sn1—C12—C17—C16	−179.07 (18)
O1—Sn1—C6—C11	155.98 (19)	Sn1—O1—C18—O2	5.1 (3)
C1—Sn1—C6—C11	27.6 (2)	Sn1—O1—C18—C19	−173.25 (14)
C12—Sn1—C6—C11	−100.6 (2)	O2—C18—C19—C20	2.0 (3)
O1—Sn1—C6—C7	−27.7 (2)	O1—C18—C19—C20	−179.78 (19)
C1—Sn1—C6—C7	−156.1 (2)	C18—C19—C20—C21	72.7 (3)
C12—Sn1—C6—C7	75.7 (2)	N2—N1—C21—C22	2.0 (3)
C11—C6—C7—C8	−1.6 (4)	N2—N1—C21—C20	−178.51 (19)
Sn1—C6—C7—C8	−178.0 (2)	C19—C20—C21—N1	3.9 (3)
C6—C7—C8—C9	0.4 (4)	C19—C20—C21—C22	−176.6 (2)
C7—C8—C9—C10	−0.1 (4)	N1—N2—C23—N3	6.1 (3)
C8—C9—C10—C11	1.0 (4)	N1—N2—C23—S1	−174.76 (16)

### Hydrogen-bond geometry (Å, °)

**Table:**

*D*—H···*A*	*D*—H	H···*A*	*D*···*A*	*D*—H···*A*
N2—H2*n*···O2^i^	0.88	2.12	2.975 (3)	163
N3—H3*n*1···O1^ii^	0.88	2.43	3.121 (3)	136
N3—H3*n*2···S1^iii^	0.88	2.54	3.389 (2)	161

Symmetry codes: (i) −*x*, −*y*+1, −*z*+1; (ii) −*x*+1, −*y*+1, −*z*+1; (iii) −*x*+1, −*y*, −*z*+1.
